# A Randomized Trial of Calcium Plus Vitamin D Supplementation and Risk of Ductal Carcinoma In Situ of the Breast

**DOI:** 10.1093/jncics/pkab072

**Published:** 2021-08-31

**Authors:** Rita Peila, Xiaonan Xue, Jane A Cauley, Rowan Chlebowski, JoAnn E Manson, Rami Nassir, Nazmus Saquib, Aladdin H Shadyab, Zhenzhen Zhang, Sylvia Wassertheil-Smoller, Thomas E Rohan

**Affiliations:** Department of Epidemiology and Population Health, Albert Einstein College of Medicine, Bronx, NY, USA; Department of Epidemiology and Population Health, Albert Einstein College of Medicine, Bronx, NY, USA; Department of Epidemiology, Graduate School of Public Health, University of Pittsburgh, Pittsburgh, PA, USA; The Lundquist Institute for Biomedical Innovation at Harbor-UCLA Medical Center, Torrance, CA, USA; Department of Medicine, Brigham and Women’s Hospital, Harvard Medical School, Boston, MA, USA; Department of Pathology, College of Medicine, Umm Al-Qura University, Saudi Arabia; College of Medicine at Sulaiman, Al Rajhi University, Sulaiman AlRajhi, Saudi Arabia; Herbert Wertheim School of Public Health and Human Longevity Science, University of California, San Diego, La Jolla, CA, USA; Division of Oncological Science, Knight Cancer Institute, Oregon Health and Science University, Portland, OR, USA; Department of Epidemiology and Population Health, Albert Einstein College of Medicine, Bronx, NY, USA; Department of Epidemiology and Population Health, Albert Einstein College of Medicine, Bronx, NY, USA

## Abstract

**Background:**

The effect of calcium plus vitamin D (CaD) supplementation on risk of ductal carcinoma in situ (DCIS) of the breast, a nonobligate precursor of invasive ductal carcinoma, is not well understood. In this secondary analysis, we examined this association in the Women’s Health Initiative CaD trial over approximately 20 years of follow-up.

**Methods:**

A total of 36 282 cancer-free postmenopausal women (50-79 years) were randomly assigned to daily (d) calcium (1000 mg) plus vitamin D (400 IU) supplementation or to a placebo. Personal supplementation with vitamin D (≤600 IU/d, subsequently raised to 1000 IU/d) and calcium (≤1000 mg/d) was allowed. The intervention phase (median = 7.1 years), was followed by a postintervention phase (additional 13.8 years), which included 86.0% of the surviving women. A total of 595 incident DCIS cases were ascertained. Hazard ratios (HRs) plus 95% confidence intervals (CIs) were calculated.

**Results:**

The intervention group had a lower risk of DCIS throughout follow-up (HR = 0.82, 95% CI = 0.70 to 0.96) and during the postintervention phase (HR = 0.76, 95% CI = 0.61 to 0.94). The group that used CaD personal supplements in combination with the trial intervention had a lower risk of DCIS compared with the trial placebo group that did not use personal supplementation (HR = 0.72, 95% CI = 0.56 to 0.91).

**Conclusions:**

CaD supplementation in postmenopausal women was associated with reduced risk of DCIS, raising the possibility that consistent use of these supplements might provide long-term benefits for the prevention of DCIS.

Ductal carcinoma in situ (DCIS) is characterized by a proliferation of epithelial cells that remain confined within the basement membrane of the breast. It is considered to be a nonobligate precursor of invasive ductal carcinoma of the breast, with an approximate estimate of 15% to 50% of cases potentially progressing to invasive breast cancer over a decade or more (1,2). An increased risk of breast cancer occurrence and mortality persists for at least 2 decades after a DCIS diagnosis (3,4). Epidemiological studies have shown that DCIS shares several risk factors with invasive breast cancer (5-7).

Experimental evidence is consistent with a potential protective effect of calcium and vitamin D in relation to breast cancer development (8-10). Furthermore, several observational epidemiological studies have suggested a possible inverse association between circulating physiological levels of calcium and vitamin D and risk of breast cancer, although the findings have not been consistent (11-15). The results of clinical trials of calcium and vitamin D supplementation in relation to breast cancer development have also been inconsistent (16-19). The Women’s Health Initiative (WHI) randomized controlled trial of calcium plus vitamin D (CaD) supplementation included approximately 36 000 postmenopausal women randomly assigned to the intervention (1000 mg calcium carbonate plus 400 IU vitamin D3 daily [d]) or to a placebo. After an intervention period of approximately 7 years, the risks of developing invasive breast cancer and DCIS were similar in the 2 trial arms (16). Subsequent analyses including outcomes that occurred up to 5 years after the end of the intervention period yielded similar results for invasive breast cancer risk but showed a statistically significant reduced risk of DCIS, particularly in the postintervention period (20). The present post hoc analysis assessed the association of CaD supplementation with risk of DCIS of the breast in the WHI trial over the entire follow-up period, including a substantially extended postintervention period of approximately 13.8 years.

## Methods

### Study Population

A detailed description of the CaD supplementation trial was presented previously (21-23). Briefly, postmenopausal women, aged 50-79 years at enrollment in WHI, and participating in 1 of the 2 hormone therapy (HT) trials or in the dietary modification (DM) trial, were eligible for inclusion in the CaD trial if they did not report hypercalcemia, renal calculi, or corticosteroid use (24). Primary outcomes of the CaD trial were hip fractures and colorectal cancer; secondary outcomes included invasive breast cancer, all cancers, coronary heart disease, and total mortality; DCIS of the breast was not a prespecified outcome (23). Personal supplementation of vitamin D (up to 600 IU/d, subsequently raised to 1000 IU/d) and calcium (up to 1000 mg/d) was allowed. A total of 36 282 women were randomly assigned to calcium (1000 mg/d of calcium carbonate) and vitamin D3 (400 IU/d) (n = 18 176) or to a placebo (n = 18 106). Of women in the CaD trial, 69.5% were in the DM trial and 44.2% were in an HT trial. The CaD trial was approved by the WHI institutional review board and by the institutional review boards at the trial sites. All women provided written informed consent. The trial was registered at clinicaltrials.gov (Identifier: NCT 00000611).

### Baseline Data Collection

At the time of enrollment in the WHI study, women completed self-administered questionnaires that sought information on demographics, medical and reproductive history, family history of cancer, and leisure-time physical activity. Height (cm) and weight (kg) were measured by clinic staff and used to determine body mass index (BMI; kg/m^2^) (23).

### Follow-up and Outcome

**Figure 1.**
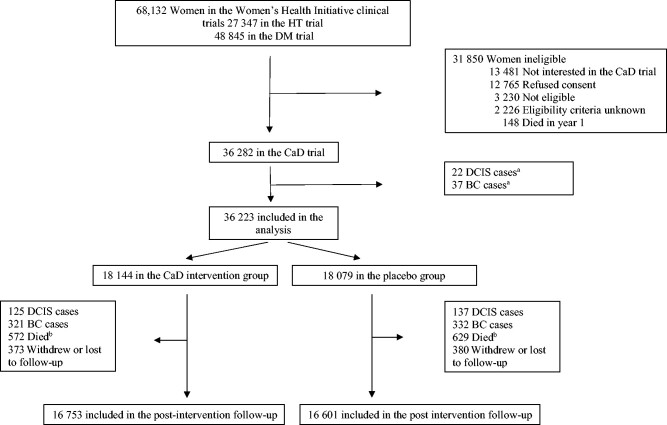
Consort diagram of participants in the Women’s Health Initiative randomized trial of calcium plus vitamin D supplementation trial showing numbers included in the analysis. ^a^Cases ascertained between the enrollment in the Women’s Health Initiative study and the enrollment in the calcium plus vitamin D trial. ^b^Among women free of DCIS of the breast and invasive breast cancer. BC = invasive breast cancer; CaD = calcium plus vitamin D intervention arm; DCIS = ductal carcinoma in situ of the breast; DM = dietary modification; HT = hormone therapy.

Protocol adherence was evaluated semiannually at each follow-up visit by weighing returned bottles of pills (25). Information on personal supplementation use was reported at baseline and at years 1, 3, and 6 after enrollment in the trial. The final clinical visits occurred between October 1, 2004, and March 31, 2005. As previously reported, at the end of the trial, vital status was known for 93.4% of participants, 76.0% still took the study pills, and 59.0% maintained 80% or greater adherence (20,23). The trial was followed by 2 extension studies (2005-2010 and 2010-2020) with the purpose of gathering additional outcome information; approximately 86.0% of the surviving trial participants provided written consent to the extended follow-up period (see Figure 1) (20).

During the intervention period, clinical breast exams and annual mammograms were protocol mandated for women enrolled in an HT trial, or biennially if in the DM trial (16). In the postintervention period, ongoing mammography was encouraged, and information on mammography was collected. Clinical outcomes were updated semi-annually, and self-reported breast cancers, both in situ and invasive, were centrally adjudicated through review of medical records and pathology reports (25).

### Statistical Analysis

Baseline characteristics of the study were compared between intervention and placebo arms using χ^2^ test for categorical variables and Wilcoxon test for continuous variables. The main objective of this study was to evaluate the association of calcium and vitamin D supplementation with the risk of DCIS of the breast over the entire follow-up period (intervention period plus postintervention period) and separately for the intervention and postintervention periods. The analyses used an intention-to treat approach, unless otherwise specified. Time at risk was calculated from the date of randomization to the CaD trial, which began an average of 1.10 (SD = 0.28) years after random assignment to the DM or the HT trial, the date of outcome occurrence, mastectomy, invasive breast cancer, death, study withdrawal, loss to follow-up, or last documented contact before March 1, 2019, whichever came first. The intervention phase of the trial ended on March 31, 2005, and this date marked the end of the time at risk for this phase and the beginning of the postintervention phase. The Kaplan-Meier method was used to estimate cumulative DCIS incidence and log-rank test *P* values to compare the survival curves. Incidence rates (IRs) per 1000 person-years were calculated at 5, 10, 15, 20, and more than 20 years of follow-up. Cox proportional hazards regression models stratified by age, DM trial randomization arm, HT trial randomization arm, and prior breast biopsy were used to estimate hazard ratios (HR) and 95% confidence intervals (CIs) for the association of CaD supplementation with DCIS risk. In addition, stratification by study phase (time dependent) was included in the model for analysis over the entire follow-up period. No violation of the proportional hazards assumption was found on the basis of examination of Schoenfeld residuals.

Our main analysis focused on the cause-specific Cox model (26,27) to examine the effect of CaD on the risk of the development of DCIS. However, because 24.0% (n = 8696) of the cohort died and 6.9% (n = 2448) was diagnosed with breast cancer during follow-up without developing DCIS, these outcomes represented competing events. Therefore, we also used the Fine and Gray subdistribution proportional hazards model to examine the effect of CaD on the incidence of DCIS in the presence of these competing events (27).

Additional analyses evaluated the robustness of the association shown in the main model (28). These included adjusting for mammogram and clinical breast exam frequency or, separately, excluding those with less than 1 year of follow-up (n = 390) or those who had a previous breast biopsy (n = 6506). To evaluate the impact of trial protocol adherence on the results, we censored women 6 months after they reported less than 80.0% adherence (n = 14 857) or less than 50.0% adherence (n = 17 717). Trial intervention arm and use of personal supplements over time (years 1, 3, and 6 post-trial initiation) were evaluated in combination, modeling personal supplement use as a time-varying covariate. Four categories of CaD intervention and/or personal supplementation were created, with the group receiving the placebo and not using personal supplementation as referent; for each interval of time defined by the clinic visits at which supplementation use was reported, participants contributed to a specific category. This analysis was adjusted for several risk factors as were reported at enrollment such as age (younger than 60, 60-69, 70 years or older), race (White, non-White), DM randomization assignment status, HT randomization assignment status, BMI (<25, 25 to <30, ≥30 kg/m^2^), years since menopause (<5, 5 to <15, ≥15 years), physical activity (no activity, 1st, 2nd, and 3rd tertile), smoking status (no/yes), and Gail risk model for breast cancer score (tertiles) (29). In addition, subgroup analyses were conducted by baseline risk factors such as age, personal CaD supplement use at enrollment, randomization assignment in the HT trials, randomization assignment in the DM trial, years since menopause, ethnicity (White, non-White), BMI, current smoking status (no, yes), physical activity, and Gail score of breast cancer risk (tertiles) (29). Multiple comparison adjustments were not performed as these were considered exploratory analyses.

Statistical analyses were conducted using Stata 16.1 (Stata Corp, College Station, TX, USA). *P* values less than .05 were considered to be statistically significant.

## Results

Baseline characteristics by intervention status were previously reported and indicated that the intervention and placebo groups were well balanced with respect to demographic characteristics, medical history, and health behaviors (16,23,30); they remained well balanced among participants in the postintervention follow-up period (Supplementary Table 1, available online). Overall, 595 DCIS cases were ascertained during the entire follow-up period (median time at risk = 18.7 years; interquartile range = 10.7-20.9 years). A total of 262 cases occurred during the intervention phase (median time at risk = 7.1 years), and 333 cases occurred during the postintervention phase (median time at risk = 13.8 years).

**Figure 2.**
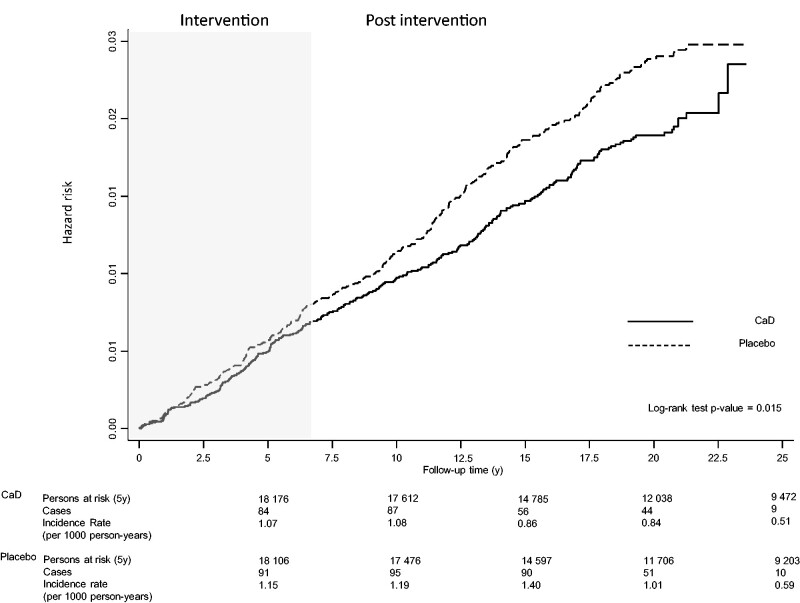
Kaplan-Meier estimates of cumulative hazards of ductal carcinoma in situ in the Women’s Health Initiative randomized trial of calcium plus vitamin D. Incidence rate per 1000 person-years was calculated at 5, 10, 15, 20, and 20 or more years of follow-up. All statistical tests were 2-sided. CaD = calcium plus vitamin D intervention arm.

Figure 2 shows Kaplan-Meier estimates of the cumulative hazard of DCIS by intervention status throughout the follow-up period. The accompanying IRs show that at each timepoint the intervention group had a lower risk of DCIS than the control group (intervention IR/1000 person-years 5, 10 , 15, 20, >20 years = 1.07, 1.08, 0.86, 0.84, and 0.51, respectively; and control IR/1000 person-years 5, 10 , 15, 20, >20 years = 1.15, 1.19, 1.40, 1.02, and 0.59, respectively). Women assigned to the intervention arm had an 18.0% reduction in risk of DCIS (HR = 0.82, 95% CI = 0.70 to 0.96) (Table 1). Similar results were obtained when the analysis was adjusted for mammogram and clinical breast exam frequency (HR = 0.80, 95% CI = 0.68 to 0.95), after exclusion of participants with a follow-up time of less than 1 year (HR = 0.81, 95% CI = 0.68 to 0.97), or after excluding women with a previous breast biopsy (HR = 0.72, 95% CI = 0.59 to 0.86). Censoring women whose adherence dropped below 80.0% or 50.0% did not impact the results (HR = 0.81 [95% CI = 0.68 to 0.96], and HR = 0.82 [95% CI = 0.69 to 0.96], respectively). Results from the Fine-Gray model also showed a decrease in the cumulative incidence of DCIS in the intervention group compared with the control group of similar magnitude (subdistribution HR = 0.82, 95% CI = 0.70 to 0.97). As previously reported (16), CaD supplementation was not associated with altered risk of DCIS during the intervention period (HR = 0.91, 95% CI = 0.71 to 1.15) (Figure 2; Supplementary Table 2, available online). In contrast, there was a statistically significant reduction in risk during the postintervention period (HR = 0.76, 95% CI = 0.61 to 0.94) (Figure 2; Supplementary Table 3, available online).

**Table 1.** Risk of ductal carcinoma in situ of the breast in women participating in the Women’s Health Initiative Calcium Plus Vitamin D trial over the entire follow-up period, overall and after various sensitivity analyses

Study population	Placebo		Calcium plus Vitamin D		HR (95% CI)^a^	*P* ^b^
	No. cases	IR/1000	No. cases	IR/1000		
Total sample	325	1.16	270	0.95	0.82 (0.70 to 0.96)	.02
Total sample^c^	325	1.16	270	0.95	0.80 (0.68 to 0.95)	.01
Excluding women with <1 year of follow-up	282	1.11	233	0.90	0.81 (0.68 to 0.97)	.02
Excluding women with previous breast biopsy	255	1.07	185	0.77	0.72 (0.59 to 0.86)	.001
Adherence 50% only	318	1.61	260	1.31	0.82 (0.69 to 0.96)	.02
Adherence 80% only	293	1.31	236	1.06	0.81 (0.68 to 0.96)	.02

a Hazard ratios and 95% confidence intervals were based on Cox proportional hazards models with stratification by age (younger than 60 years, 60 to younger than 70 years, 70 years or older), dietary modification trial participation, hormone replacement treatment trial participation, and prior breast biopsy. CI = confidence interval; HR = hazard ratio; IR/1000 = incidence rate per 1000 person-years.

b Two-sided χ^2^ test.

c Analysis adjusted for mammogram and clinical breast exam frequency.

**Figure 3.**
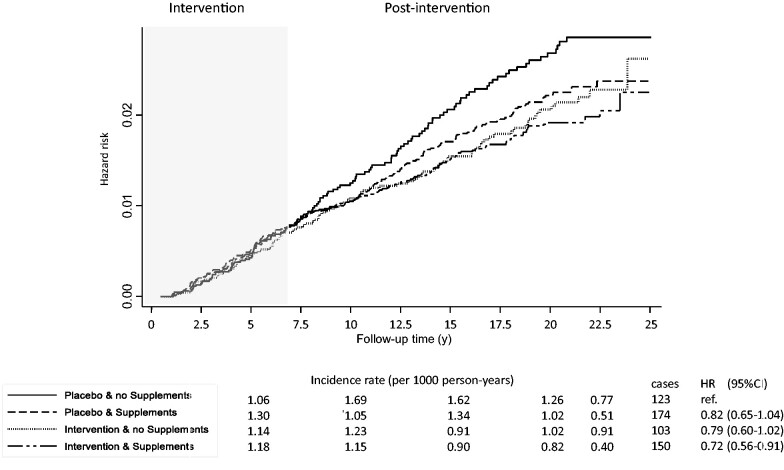
Kaplan-Meier estimates of cumulative hazards of ductal carcinoma in situ by trial arm assignment and reported personal use of calcium plus vitamin D supplements over time. Hazard ratios (HRs) and 95% confidence intervals (CIs) were adjusted for age, race, body mass index, hormone therapy enrollment and randomization, dietary modification enrollment and randomization, years since menopause, physical activity, and Gail score for breast cancer risk. Incidence rate per 1000 person-years was calculated at 5, 10, 15, 20, and 20 or more years of follow-up.

Interval time IRs, cumulative incidences, and hazard ratios in groups defined by intervention status and use of personal supplementation of calcium and vitamin D are shown in Figure 3. Compared with women in the placebo group who did not take personal supplementation throughout the trial period, those in the intervention group using personal supplementation had lower IRs during the postintervention period and reduced DCIS risk (HR = 0.72, 95% CI = 0.56 to 0.91), whereas in the other 2 groups (personal supplementation only and intervention supplementation only), the IRs and hazard ratios were intermediate in magnitude and did not reach statistical significance.

**Figure 4.**
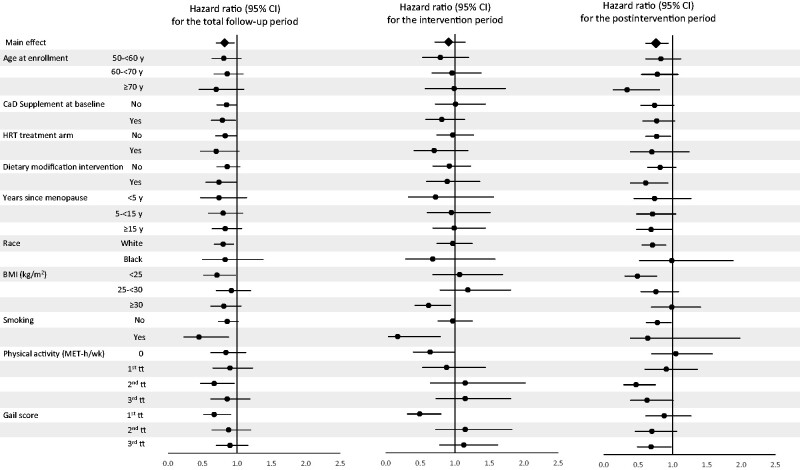
Hazard ratios (HR) for risk of ductal carcinoma in situ of the breast associated with supplemental calcium plus vitamin D (CaD) according to selected baseline characteristics. The error bars represent the 95% confidence intervals (CIs). The analyses were stratified by age, hormone therapy trial arm participation, dietary trial arm participation, and previous breast biopsy. The analyses were stratified by trial phase (time-dependent). Physical activity (metabolic equivalent-hours/week [MET-h/wk]) tertiles (tt) cutoffs: tt0 = 0; tt1 = 0.50-5.33; tt2 = 5.38-14.17; tt3 = 14.25-134.17. Gail model risk of breast cancer tertiles cutoffs: tt1 = 0.37-1.24; tt2 = 1.25-1.74; tt3 = 1.75-12.97. BMI = body mass index; CaD = calcium plus vitamin D; HRT = hormone replacement therapy.

The results of subgroup analyses are summarized in Figure 4 and presented in detail in Supplementary Tables 2-4 (available online). Over the entire follow-up period, in the subgroups defined by various potential risk factors for DCIS, there was some suggestion of a reduction in risk, albeit mostly statistically nonsignificant (Figure 4; Supplementary Table 4, available online). Analyses limited to the intervention phase showed that most of the associations were close to the null (Figure 4; Supplementary Table 2, available online), although there was some evidence for heterogeneity in the associations by subgroup for BMI (*P*_interaction_ = .046), recreational physical activity (*P*_interaction_ = .047), smoking (*P*_interaction_ = .03), and Gail model score (*P*_interaction_ = .001). Subgroup analyses of the postintervention phase showed reduced DCIS risk associated with the intervention in most of the subgroups with the exception of BMI (*P*_interaction_ = .02), levels of physical activity (*P*_interaction_ = .03), and Gail model score (*P*_interaction_ = .007) (Figure 4; Supplementary Table 3, available online).

## Discussion

In this secondary analysis of data from the WHI CaD supplementation trial covering an extended postintervention follow-up period, we found that the intervention was associated with a reduced risk of developing DCIS of the breast. These results remained statistically significant after the exclusion of participants with a follow-up of less than 1 year or of those who had a breast biopsy before the beginning of the trial. Similar results were also found when the analysis was restricted to those with a high or medium level of adherence to the study medication.

The present study builds on findings from previous WHI reports on the association of calcium and vitamin D with DCIS. Results from the intervention period of this trial showed no difference in DCIS risk between the 2 trial arms (16). The lack of effect observed through the intervention phase of the trial might have been due to the relatively low number of cases (n = 262) during this period. In a subsequent report from this trial with 436 DCIS cases over an extended follow-up (intervention plus a median of 4.9 years of postintervention follow-up), there was a statistically significant reduction in DCIS risk during the total follow-up (HR = 0.82, 95% CI = 0.68 to 0.99) and the postintervention period (HR = 0.63, 95% CI = 0.45 to 0.88) (20). The present analysis extended the postintervention period and confirmed a statistically significant reduction in risk of DCIS associated with calcium and vitamin D supplementation. In addition, we found that the greatest reduction in risk was observed in women who were in the intervention arm and also used personal CaD supplementation during the trial period. Given the relatively low dosage used in the trial protocol compared with current recommendations (31), it is possible that the additional personal CaD intake in this group contributed to the reduction in DCIS risk. However, given that this analysis was based on comparison of nonrandomized groups, we cannot exclude the possibility that women who used CaD personal supplements might also have adopted healthier dietary and lifestyle habits (32) that protected them against DCIS. A previous analysis of the association of the intervention with DCIS risk during the trial period and stratified by baseline personal supplementation did not find a statistically significant difference between the intervention and placebo groups (33). Our analysis included the intervention and the postintervention periods with extensive follow-up and took into account changes in the use of CaD personal supplementation over the intervention phase. Nevertheless, the results of the study are based on post hoc analyses and should be regarded as hypothesis generating.

In the present study, with extended follow-up, we performed subgroup analyses defined by several potential risk factors for DCIS over the entire follow-up period and separately for the intervention and postintervention phases of the trial. The results of the subgroup analyses restricted to the intervention phase showed that women in the intervention arm with a BMI of 30 kg/m^2^ or higher, or no physical activity, or with low breast cancer risk based on the Gail score had a reduced risk of DCIS. The subgroup analyses focused on the postintervention period showed that the trial intervention was associated with a statistically significant reduction in DCIS risk among women who were older, had a normal BMI (<25kg/m^2^), did not use hormone therapy, were enrolled in the DM intervention arm, or had a higher level of physical activity (6,34-36). Among women with high breast cancer risk based on the Gail score, those in the intervention group also had a lower risk than those in the placebo group. The reasons for the differences in the patterns of associations in some of the subgroup analyses by trial phase are not completely clear. Overall, these findings, although potentially providing further insight into the association between CaD supplementation and DCIS risk over time, were exploratory and need to be interpreted with caution.

Several lines of research have focused on the potential role of calcium and vitamin D in the development of breast cancer. In vitro and in vivo experimental studies have suggested a role for vitamin D and calcium in preventing breast cancer development and progression (8-10,37). Studies in rodents have shown that increasing serum calcium concentration reduces cell proliferation and induces differentiation and apoptosis (38). Similarly, vitamin D induces inhibition of cancer cell proliferation, modulation of proapoptotic and anti-angiogenic mechanisms, downregulation of inflammatory mechanisms, and immune response modulation in animal models (39,40). Observational studies have focused on the association of serum and dietary calcium (11) and vitamin D (12,41) with the risk of breast cancer and have shown some inconsistencies (42). To the best of our knowledge, the WHI CaD trial is the only study that has reported on the association of CaD supplementation with DCIS separately from that with invasive breast cancer (16,20,33). Three nested case-control studies that examined the association between circulating levels of vitamin D and breast cancer risk included DCIS cases but did not report results separately for DCIS and invasive breast cancer (43-45). A recent clinical trial of CaD supplementation administered for 1 year reported a statistically nonsignificant reduction in breast cancer risk during a 4-year follow-up period but observed only a few DCIS cases during this time (19). Future studies may help clarify the role of calcium and vitamin D in breast cancer etiology (46,47), however, they will require large sample sizes and many years of follow-up to evaluate DCIS risk.

The results of the present analyses were based on data from a large, double-blind, placebo-controlled, randomized trial. The major strengths and weaknesses of the trial have been discussed previously (16,20-22). Of particular relevance here, the trial design included collection of information on breast cancer risk factors and semiannual monitoring of protocol adherence, which remained high over the duration of the trial. Protocol-mandated annual or biennial mammograms allowed the detection of DCIS cases, which would otherwise have remained undiagnosed. The extensive follow-up available with a relatively high number of DCIS cases ascertained provided sufficient statistical power for the main analyses. Longitudinal data on personal supplement use and on adherence to the intervention throughout the trial were available for the majority of the participants and were included in the analyses. We used a cause-specific Cox model to examine the mechanistic relationship between intervention and DCIS and the competing risk model to examine the effect of intervention on cumulative incidence of DCIS taking into account the intervention effects on competing events such as invasive breast cancer and death. The results of these 2 models were similar, suggesting that the lower cumulative incidence of DCIS in the intervention group obtained from the competing risk model was largely attributed to the effect of CaD on the risk of DCIS and not on the risk of competing events.

One of the main limitations of the study was the inability to analyze the association of calcium and vitamin D separately. Furthermore, the present study did not take into account calcium and vitamin D intake from dietary sources as this was not updated as frequently as information on personal supplementation. We did not have reports on personal supplementation during the postintervention phase, which might have contributed to circulating levels of calcium and vitamin D over this period. Available data on postintervention mammogram screening indicated an overall reduction in the frequency, potentially causing underascertainment of DCIS cases; however, the frequency of screening remained similar between the 2 arms of the trial, limiting potential selection bias during this period. Finally, we did not have access to information on the hormone-receptor status of the DCIS cases.

In conclusion, the results of this study suggest that CaD supplementation reduces the risk of DCIS of the breast. Given that DCIS is a breast cancer precursor, these findings raise the possibility that CaD supplementation might ultimately reduce breast cancer risk by acting at a relatively early stage in the natural history of the disease.

## Funding

Dr Rohan is supported in part by the Breast Cancer Research Foundation (BCRF-20-140).

## Notes

**Role of the funder:** The funders did not play a role in the design of the study; the collection, analysis, and interpretation of the data; the writing of the manuscript; or the decision to submit the manuscript for publication.

**Disclosures:** The authors have no disclosures.

**Author contributions:** Conceptualization—TER. Data curation, formal analysis, and investigation—RP. Methodology—RP, XX:. Writing—original draft—RP, TER. Writing—rewriting & editing—all authors.

**Acknowledgements:** We thank the Women’s Health Initiative investigators, staff, and the trial participants for their outstanding dedication and commitment. A full list of all the investigators who have contributed to Women’s Health Initiative science appears at: https://www.whi.org/researchers/Documents%20%20Write%20a%20Paper/WHI%20Investigator%20Long%20List.pdf.

## Supplementary Material

pkab072_Supplementary_Data

## Data Availability

Data is available through the WHI online resource, https://www.whi.org/researchers/data/Pages/Home.aspx, and the WHI remains funded indefinitely through BioLINCC, https://biolincc.nhlbi.nih.gov/studies/whi_ctos/.
