# Supplementary material for: A Randomized Trial of Calcium Plus Vitamin D Supplementation and Risk of Ductal Carcinoma In Situ of the Breast
Source: JNCI Cancer Spectr. 2021 Aug 31;5(4):pkab072. doi: 10.1093/jncics/pkab072 (PMC8406436; doi:10.1093/jncics/pkab072)
Supplement: pkab072_Supplementary_Data [file pkab072_supplementary_data.pdf]

Supplementary Table 1. Baseline characteristics of Women's Health Initiative calcium plus vitamin D participants in the post-intervention extension study, by randomization assignment

Characteristics	Placebo N = 16,601	Calcium plus vitamin D N = 16,753	*P*
Age group, No. (%)			
50-<60 y	6,187 (37.3)	6,274 (37.5)	0.93[a]
60-<70 y	7,597 (45.8)	7,656 (45.7)	
≥70 y	2,817 (17.0)	2,823 (16.9)	
Race/ethnicity, No. (%)			
White	13,863 (83.5)	13,901 (83.0)	0.39[a]
Black	1,494 (9.0)	1,532 (9.1)	
Hispanic	651 (3.9)	716 (4.3)	
Others	593 (3.6)	604 (3.6)	
Education, No. (%)			
None to some high school	823 (5.0)	887 (5.3)	0.49[a]
High school diploma	4,832 (29.1)	4,805 (28.7)	
School after high school	10,841 (65.3)	10,957 (65.4)	
HRT treatment, No. (%)			
No	12,877 (77.6)	13,052 (77.9)	0.68[a]
Estradiol only	1,404 (8.5)	1,415 (8.5)	
Estradiol plus progestin	2,320 (14.0)	2,286 (13.7)	
Dietary intervention, No. (%)			
No	12,157 (73.2)	12,364 (73.8)	0.24[a]
Yes	4,389 (26.2)	4,444 (26.8)	
Calcium plus vitamin D personal supplement, No. (%)			
No	9,851 (59.3)	10,097 (60.3)	0.08[a]
Yes	6,750 (40.7)	6,656 (39.7)	
Years since menopause, No. (%)			
<5 y	1,775 (10.7)	1,815 (10.8)	0.93[a]
5-<15 y	4,813 (29.0)	4,810 (28.7)	
≥15 y	7,274 (43.8)	7,370 (44.0)	
BMI, No. (%)			
<25 kg/m^2	4,468 (26.9)	4,382 (26.2)	0.19[aa]
25-<30 kg/m^2	5,981 (36.0)	5,989 (35.8)	
≥30 kg/m^2	6,063 (36.5)	6,298 (37.6)	
Current smoking, No. (%)			
No	15,410 (92.8)	15,499 (92.5)	0.28
Yes	1,191 (7.2)	1,254 (7.5)	
Physical activity, No. (%)			
No	4,339 (26.2)	4,335 (25.9)	0.56[a]
1st tertile	3,986 (24.0)	4,058 (24.3)	
2nd tertile	3,997 (24.1)	4,121 (24.6)	
3rd tertile	4,265 (25.7)	4,223 (25.2)	
Gail 5-year risk of breast cancer, No. (%)			
1st tertile	5,799 (34.9)	5,888 (35.2)	0.89[a]
2nd tertile	5,464 (32.9)	5,478 (32.7)	
3rd tertile	5,338 (32.2)	5,387 (32.2)	
Calcium personal supplement, mg[c], No. (median)			
Baseline	9,226 (573.1)	9,090 (585.4)	0.14[b]
Year 1	9,652 (582.2)	9,563 (579.7)	0.74[b]

Year 3	9,821 (540.7)	9,659 (530.9)	0.19 [b]
Year 6	10,566 (548.5)	10,716 (546.0)	0.74 [b]
Vitamin D person supplement, IU[c], No. (median)			
Baseline	7,981 (395.2)	7,798 (395.2)	0.90 [b]
Year 1	8,457 (403.2)	8,399 (403.2)	0.96 [b]
Year 3	8,983 (411.6)	8,974 (409.2)	0.34 [b]
Year 6	10,099 (429.2)	9,971 (432.8)	0.19 [b]

[a] Two-sided χ^2 test.

[b] Two-sided Wilcoxon rank sum test.

[c] Values among women using personal supplements.

Supplementary Table 2. Incidence rates and hazard ratios (95% confidence intervals) for the association of calcium plus vitamin D with risk of ductal carcinoma in situ of the breast during the intervention period

	Placebo		Ca and Vit.D			
Intervention period	No. cases	IR/1,000	No. cases	IR/1,000	HR (95% CI) [a]	*P* [b]
Total sample	137	1.26	125	1.14	0.91 (0.71-1.15)	0.42
Age group, years						
50-<60	51	1.21	41	0.96	0.79 (0.53-1.20)	0.27
60-<70	61	1.26	59	1.22	0.96 (0.67-1.38)	0.83
≥70	25	1.38	25	1.37	0.99 (0.57-1.73)	0.99
Interaction term age group*CaD *P* [b]						0.48
Ca and Vit D personal supplement						
No	82	1.27	82	1.24	0.98 (0.72-1.33)	0.88
Yes	55	1.29	43	1.03	0.79 (0.53-1.18)	0.26
Interaction term CaD personal supplement*CaD *P* [b]						0.42
HRT treatment						
No	104	1.23	102	1.20	0.97 (0.74-1.27)	0.83
Yes	33	1.36	23	0.95	0.70 (0.41-1.19)	0.19
Interaction term HRT treatment*CaD *P* [b]						0.29
Dietary intervention						
No	90	1.14	84	1.04	0.92 (0.68-1.23)	0.56
Yes	47	1.41	41	1.59	0.89 (0.59-1.36)	0.59
Interaction term dietary intervention*CaD *P* [b]						0.91
Years since menopause						
<5	15	1.27	11	0.91	0.72 (0.33-1.56)	0.40
5-<15	37	1.20	35	1.14	0.95 (0.60-1.51)	0.83
≥15	56	1.22	56	1.21	0.99 (0.68-1.44)	0.96
Interaction term years since menopause*CaD *P* [b]						0.98
Ethnicity						
White	114	1.25	110	1.21	0.97 (0.74-1.25)	0.78
Black	13	1.34	9	0.91	0.68 (0.29-1.58)	0.37
Interaction term ethnicity*CaD *P* [b]						0.21
BMI, kg/m^2						
<25	36	1.23	38	1.32	1.07 (0.68-1.69)	0.77
25-<30	41	1.05	49	1.25	1.19 (0.79-1.81)	0.40
≥30	59	1.49	38	0.93	0.62 (0.42-0.94)	0.02
Interaction term BMI*CaD *P* [b]						0.046
Current smoking						
No	124	1.25	121	1.21	0.97 (0.76-1.25)	0.81
Yes	11	1.36	2	0.24	0.17 (0.04-0.79)	0.02
Interaction term smoking*CaD *P* [b]						0.03

Physical activity						
No activity	47	1.55	30	0.98	0.64 (0.40-1.00)	0.05
1 tertile	33	1.28	29	1.12	0.88 (0.53-1.44)	0.60
2 tertile	22	0.86	26	0.99	1.15 (0.65-2.02)	0.64
3 tertile	35	1.30	40	1.49	1.15 (0.73-1.81)	0.55
Interaction term physical activity*CaD *P* [b]						0.047
Gail score risk for breast cancer						
1 tertile	50	1.36	25	0.67	0.49 (0.31-0.80)	0.004
2 tertile	33	0.91	38	1.06	1.15 (0.72-1.83)	0.55
3 tertile	54	1.56	62	1.76	1.13 (0.78-1.62)	0.52
Interaction Gail breast cancer risk*CaD *P* [b]						0.001

[a] Proportional hazard model stratified by age, hormone replacement treatment trial randomization, dietary modification trial randomization, prior biopsy.

[b] *P* values were calculated using 2-sided Wald test.

Abbreviations: IR, incidence rate; PY, person-years; HR, hazard ratio; CI, confidence interval.

Supplementary Table 3. Incidence rates and hazard ratios (95% confidence intervals) for the association of calcium plus vitamin D with risk of ductal carcinoma in situ of the breast during the post-intervention period

	Placebo		Ca and Vit.D			
Post intervention period	No. cases	IR/1,000	No. cases	IR/1,000	HR (95% CI)	*P*[a]
Total sample [b]	188	1.04	145	0.79	0.76 (0.61-0.94)	0.01
Age-group						
50-<60	90	1.23	77	1.02	0.83 (0.61-1.12)	0.23
60-<70	78	0.93	61	0.72	0.78 (0.55-1.08)	0.14
≥70	20	0.85	7	0.29	0.34 (0.14-0.81)	0.01
Interaction term age group*CaD *P*						0.13
Ca and Vit D personal supplement						
No	86	1.08	67	0.80	0.74 (0.54-1.02)	0.06
Yes	102	1.01	78	0.78	0.77 (0.57-1.03)	0.08
Interaction term CaD personal supplement*CaD *P*						0.46
HRT treatment						
No	160	1.13	125	0.86	0.77 (0.61-0.97)	0.03
Yes	28	0.73	20	0.51	0.70 (0.39-1.24)	0.22
Interaction term HRT*CaD *P*						0.79
Dietary intervention						
No	133	1.01	112	0.82	0.82 (0.64-1.05)	0.11
Yes	55	1.11	33	0.67	0.61 (0.39-0.93)	0.02
Interaction term dietary intervention*CaD *P*						0.24
Years since menopause						
<5	31	1.45	24	1.08	0.74 (0.44-1.27)	0.28
5-<15	59	1.05	42	0.74	0.71(0.48-1.05)	0.09
≥15	69	0.74	49	0.49	0.69 (0.48-0.99)	0.049
Interaction term years since menopause*CaD *P*						0.46
Ethnicity						
White	161	1.04	115	0.73	0.71 (0.56-0.90)	0.004
Black	18	1.27	19	1.26	0.99 (0.52-1.88)	0.97
Interaction term ethnicity*CaD *P*						0.30
BMI, kg/m^2						
<25	57	1.14	28	0.56	0.49 (0.31-0.77)	0.002
25-<30	71	0.86	55	0.63	0.76 (0.54-1.09)	0.13
≥30	60	0.94	62	0.93	0.99 (0.69-1.41)	0.96
Interaction term BMI*CaD *P*						0.02
Current smoking						
No	168	0.86	134	0.79	0.78 (0.62-0.98)	0.04
Yes	16	1.41	11	0.9	0.64 (0.30-1.98)	0.26
Interaction term smoking*CaD *P*						0.68

Physical activity						
No activity	44	0.96	47	1.00	1.05 (0.70-1.58)	0.82
1 tertile	48	1.12	45	1.02	0.91 (0.60-1.36)	0.63
2 tertile	51	1.16	25	0.54	0.47 (0.29-0.75)	0.002
3 tertile	45	0.94	28	0.59	0.63 (0.39-1.01)	0.06
Interaction term physical activity*CaD *P*						0.03
Gail score risk for breast cancer						
1 tertile	60	1.03	55	0.91	0.88 (0.61-1.27)	0.50
2 tertile	54	0.90	38	0.63	0.70 (0.46-1.06)	0.09
3 tertile	74	1.35	52	0.93	0.69 (0.49-0.98)	0.04
Interaction Gail breast cancer risk*CaD *P*						0.007

[a] *P* values were calculated using 2-sided Wald test.

[b] Proportional hazard model stratified by age, hormone replacement treatment trial randomization, dietary modification trial randomization, prior biopsy.

Abbreviations: IR, incidence rate; PY, person-years; HR, hazard ratio; CI, confidence interval.

Supplementary Table 4. Incidence rates and hazard ratios (95% confidence intervals) for the association of calcium plus vitamin D with risk of ductal carcinoma in situ of the breast over the entire follow-up period

	Placebo		Ca and Vit.D			
Overall follow-up	No. cases	IR/1,000	No. cases	IR/1,000	HR (95% CI)	*P*[a]
Total sample [b]	325	1.12	270	0.92	0.82 (0.70-0.96)	0.02
Age-groups						
50-<60	141	1.22	118	1.00	0.81 (0.64-1.04)	0.10
60-<70	139	1.05	120	0.90	0.86 (0.67-1.09)	0.22
≥70	45	1.08	32	0.75	0.70 (0.45-1.10)	0.13
Interaction term age group*CaD *P*						0.71
Ca and Vit D personal supplement						
No	148	1.14	132	0.97	0.85 (0.71-1.00)	0.18
Yes	177	1.11	138	0.87	0.79 (0.63-0.98)	0.04
Interaction term supplement use*CaD *P*						0.62
HRT treatment arm						
No	264	1.17	227	0.99	0.83 (0.69-0.99)	0.06
Yes	61	0.68	43	0.67	0.70 (0.47-1.03)	0.07
Interaction term HRT treatment*CaD *P*						0.32
DM intervention arm						
No	223	1.06	196	0.91	0.86 (0.71-1.04)	0.12
Yes	102	1.29	74	0.95	0.74 (0.55-0.99)	0.04
Interaction term DM intervention*CaD *P*						0.40
Years since menopause						
<5	46	1.38	35	1.02	0.74 (0.47-1.14)	0.17
5-<15	96	1.11	77	0.88	0.80 (0.59-1.08)	0.15
≥15	125	1.05	105	0.87	0.83 (0.64-1.07)	0.15
Interaction term years since menopause*CaD *P*						0.50
Ethnicity						
White	275	1.12	225	0.91	0.80 (0.67-0.95)	0.01
Black	31	1.30	28	1.12	0.83 (0.50-1.38)	0.47
Interaction term ethnicity*CaD *P*						0.23
BMI, kg/m^2						
<25	93	1.17	66	0.84	0.71 (0.52-0.98)	0.04
25-<30	112	1.07	104	0.98	0.92 (0.70-1.20)	0.54
≥30	119	1.15	100	0.93	0.81 (0.62-1.06)	0.12

Interaction BMI groups*CaD *P*						0.69
Current smoking						
No	292	1.09	255	0.94	0.86 (0.73-1.02)	0.09
Yes	27	1.39	13	0.63	0.45 (0.23-0.88)	0.02
Interaction current smoking status*CaD *P*						0.07
Physical activity						
No activity	91	1.19	77	1.00	0.84 (0.62-1.13)	0.25
1 tertile	81	1.18	74	1.06	0.90 (0.65-1.23)	0.49
2 tertile	73	1.05	51	0.70	0.67 (0.47-0.96)	0.03
3 tertile	80	1.07	68	0.92	0.86 (0.62-1.19)	0.35
Interaction physical activity*CaD *P*						0.79
Gail score risk for breast cancer						
1 tertile	116	1.14	82	0.78	0.67 (0.52-0.91)	0.01
2 tertile	84	0.87	74	0.77	0.88 (0.64-1.20)	0.41
3 tertile	125	1.38	114	1.24	0.90 (0.70-1.16)	0.42
Interaction physical activity*CaD *P*						0.001

[a] *P* values were calculated using 2-sided Wald test.

[b] Proportional hazard model stratified by age, hormone replacement treatment trial randomization, dietary modification trial randomization, prior biopsy.

Abbreviations: IR, incidence rate; PY, person-years; HR, hazard ratio; CI, confidence interval.
